# Experimental Researches on Possibility of Migration of Metallic Projectiles

**Published:** 1918-12

**Authors:** 


**Experimental Researches on the Possibility of the Migration of Metallic Projectiles in the Circulatory System.** By MM. Ch. ACHARD and L. BINET. *Bull. de l'Académie de Médecine,* July 16, 1918.

Very many cases of migration of projectiles in the circulatory system have been studied lately. The projectile can either move *within the arteries*, for instance, be carried away by the blood from the heart into the aorta and from this into some periphereal artery, or *within the veins*, and move from the periphery to the heart or from this to the periphery. In some cases the projectile moves in a certain direction, and the blood flows in the opposite one.

Tests have been carried out with shot (spherical lead balls, 2 mm. in diameter) introduced into the circulatory system of dogs.

1° *Migration in the arteries.* The blood pressure is so strong that the shot, if introduced into a large artery, moves rapidly into a peripherical vessel, even if this movement is contrary to the action of the weight.

2° *Migration in the veins.* Three kinds of experiments were carried out, according to the position of the animal; this was either horizontal, or inclined with its head downwards, or inclined with its head upwards. The projectile is pushed into the veins by injecting simultaneously 5 cc. of normal saline solution in the vessel.

The blood pressure is not strong enough to carry away the projectiles against the action of weight. The metallic projectiles move according to the position of the body. The movement depends also on the volume, weight, shape, and outline of the projectile.

Two opposite actions interfere: the blood pressure, and a force of friction. The action of weight is to be added to one or the other force according to the position of the body.

Small projectiles, as used in the experiments, had a tendency of penetrating into the deverticula of the veins.

These results prove the necessity of localizing an intravascular projectile only just before its removal is attempted.

